# From Culture-Negative to DNA-Positive: The Molecular Revolution in Infective Endocarditis Diagnosis

**DOI:** 10.3390/pathogens14060518

**Published:** 2025-05-23

**Authors:** Myeongji Kim, Madiha Fida, Omar M. Abu Saleh, Nischal Ranganath

**Affiliations:** Division of Public Health, Infectious Diseases, and Occupational Medicine, Mayo Clinic, Rochester, MN 55905, USA; fida.madiha@mayo.edu (M.F.); abusaleh.omar@mayo.edu (O.M.A.S.); ranganath.nischal@mayo.edu (N.R.)

**Keywords:** infective endocarditis, molecular diagnostics, polymerase chain reaction, metagenomic sequencing, broad-range polymerase chain reaction, microbial cell-free DNA

## Abstract

Infective endocarditis (IE) remains a diagnostic challenge, particularly in cases where microbiological diagnosis is not established. Advances in molecular diagnostics have expanded the ability to identify causative pathogens beyond traditional culture-based methods. This review explores the role of molecular assays, including pathogen-specific PCR, multiplex PCR, broad-range PCR, and shotgun metagenomic sequencing, in diagnosing IE. These molecular techniques enhance pathogen detection, especially in patients with prior antibiotic exposure, and improve diagnostic accuracy in culture-negative IE. Broad-range PCR assays and metagenomic sequencing offer the untargeted detection of a wide spectrum of organisms. Despite their advantages, limitations such as availability, interpretation challenges, and a lack of antimicrobial susceptibility testing remain. A multimodal approach integrating molecular diagnostics with conventional methods is essential to optimize patient management. Further research is needed to refine diagnostic algorithms and improve cost-effectiveness in clinical practice.

## 1. Introduction

Diagnostic criteria for infective endocarditis (IE) involve clinical signs and symptoms, imaging, microbiology, pathology, and intraoperative findings [1]. The mainstay of microbiological diagnosis has been bacterial and fungal cultures of blood and native or prosthetic valve tissues. However, 10–20% of IE cases transpire to be culture-negative, posing challenges to optimal therapeutic interventions [2,3]. Advances in serologic and molecular diagnostics have complemented traditional cultures to overcome these challenges in culture-negative IE (CNIE). In keeping, the updated 2023 Duke-International Society for Cardiovascular Infectious Diseases (ISCVID) criteria incorporated various serologic and molecular diagnostics in their pathologic and microbiologic criteria [1]. Pathologic criteria for definite endocarditis now acknowledge polymerase chain reaction (PCR) or other nucleic-acid-based testing as valid methods to detect microorganisms in cardiac tissue, vegetation, cardiac prosthesis, and arterial embolus [1]. Microbiologic major criterion A.2.i, a new criterion, incorporates PCR or other nucleic-acid-based tests for *Coxiella burnetii, Bartonella* species, and *Tropheryma whipplei* in blood [1]. Microbiologic minor criterion E.2 also endorses PCR or other nucleic-acid-based tests from a sterile body site other than cardiac tissue, cardiac prosthesis, or arterial embolus [1]. Here, we review different molecular diagnostic assays, their advantages and limitations, the importance of a multimodal approach, and future directions in the field of microbiologic diagnosis in IE.

## 2. Pathogen-Specific Polymerase Chain Reaction

Pathogen-specific PCR assays have traditionally been utilized in combination with serologic studies to detect microorganisms that are not readily recoverable via conventional bacterial or fungal cultures. Examples include PCR assays for *C. burnetii*, *Bartonella* spp., and *T. whipplei*, all of which can be performed using both blood and cardiac tissues. 

The reported sensitivity of *C. burnetii* PCR assays for Q fever endocarditis ranges from 33% to 69% in blood specimens and 25% to 75% in tissue specimens [4,5,6,7]. The sensitivity is lower if the diagnostic test is delayed while on antibiotic therapy, and a tissue PCR performs notably better than a blood PCR [4,5,6,7]. PCR assays for *Bartonella* spp. appear to have a better diagnostic performance because, in one study, the sensitivity of a *Bartonella-*specific real-time PCR (RT-PCR) reached 92% for valvular biopsies [8]. The *Bartonella-*specific RT-PCR of serum had a lower sensitivity of 50% [8]. Similarly, for *T. whipplei*, a dedicated PCR using cardiac tissue is considered to be the most sensitive test, with a sensitivity of 72% reported in a review by McGee et al. There is no study directly comparing the performance of dedicated PCR and broad-range PCR assays [9]. Similar to *C. burnetii* and *Bartonella* spp., the serum PCR for *T. whipplei* performs worse than the tissue PCR, with a sensitivity of 31.2% [10].

With the limited sensitivity and availability of PCR testing, serology has remained the mainstay of diagnostic testing for infection caused by these organisms. However, this paradigm is evolving as broad-range PCR assays and metagenomic sequencing have become available, as discussed below. Thus far, there is no study directly comparing the diagnostic performance of pathogen-specific PCR assays with broad-range PCR assays or metagenomic sequencing, either in blood or tissue. Therefore, further research is needed to elucidate whether performing pathogen-specific PCR testing has an additive value to broad-range PCR testing or metagenomic sequencing.

## 3. Multiplex Real-Time Polymerase Chain Reaction

Multiplex PCR refers to a PCR assay performed with a prespecified set of primers, enabling the detection of a panel of bacterial and fungal organisms. The number of species that can be detected by the assay is often around 25 to 30 but it varies between commercial assays depending on the design of primer sets. Currently, commercially available multiplex PCR assays are used for the identification of pathogens where there is growth in blood culture bottles. Their use in the detection of pathogens in tissue is off-label and investigational. Fernández et al. performed commercial multiplex PCR testing on cardiac valve tissue explanted from 15 patients with definite IE [11]. Among the 15 cases, 10 had negative heart valve cultures, whereas all had positive multiplex PCR assays for heart valves, and organisms detected included *Streptococcus* spp., *Staphylococcus aureus,* coagulase-negative *Staphylococcus,* and *Enterococcus faecalis* [11]. Similarly, a study by Bast et al. found that six culture-negative valve tissues had positive multiplex PCR assays, which were concurrently confirmed by 16S ribosomal RNA (rRNA) gene sequencing [12]. Leli et al. demonstrated drastically improved sensitivity in tissue multiplex PCR assays (95%) versus tissue culture (15%) [13]. In these studies, tissue culture-negative cases were attributed to antimicrobial exposure prior to cardiac surgery, demonstrating a superiority of PCR assays over conventional tissue culture for pathogen detection in the patients who had already been treated with antimicrobials.

Another advantage of multiplex real-time PCR assays for cardiac tissue is the faster time to obtain a result than tissue culture [12]. Bast et al. reported a mean turnaround time of 2.3 h for multiplex PCR assays, which is significantly shorter than those for culture or broad-range PCR assays [12]. This fast turnaround time could also be useful in the case of negative results, not just in the case of positive detection. Given that conventional cultures usually takes 5 days to be finalized as no growth, a fast turnaround time combined with the high negative predictive value of multiplex PCR assays could aid clinicians with timely diagnosis and the initiation of appropriate treatment.

In contrast to blood or tissue cultures, multiplex PCR assays cannot provide full antimicrobial susceptibility results. However, several commercial assays can detect important resistance genes such as *mecA, vanA, vanB,* and carbapenemase genes. Given the fast turnaround time, this information can guide the clinician with initial antibiotic treatment decisions for IE.

Multiplex PCR assays are limited in their number of detectable species and ability to discern certain genera to a species level. Although most commercial multiplex PCR assays detect common pathogens for IE (i.e., streptococci, staphylococci, and enterococci), which account for 80 to 90% of all IE cases [14,15], they will not detect other organisms such as HACEK group microorganisms (*Haemophilus* species, *Aggregatibacter actinomycetemcomitans, Cardiobacterium hominis*, *Eikenella corrodens*, and *Kingella kingae*), *C. burnetii*, *Bartonella* spp., *T. whipplei*, *Cutibacterium* spp., and *Corynebacterium* spp. In addition, conventional multiple PCR assays identify only a few streptococci and coagulase-negative staphylococci to a species level. 

With these limitations, as mentioned before, multiplex PCR assays for tissue have only been utilized for research purposes. Broad-range PCR assays, rather than multiplex PCR assays, should be performed on tissue if broad-range PCR testing is available. The strengths of broad-range PCR assays for tissue are discussed in detail in the next section. Currently, the use of multiplex PCR assays for blood is limited to the identification of species when the blood culture bottle signals growth, which is not applicable in CNIE. The only molecular testing that is used for the direct blood detection of pathogens is plasma microbial cell-free DNA (mcfDNA) sequencing, as discussed below. 

## 4. Broad-Range Polymerase Chain Reaction and Sequencing

16S rRNA universally exists in the domain of bacteria, like 18S rRNA and 28S rRNA exist in the domain of fungi. In these rRNA gene sequences, there are hypervariable regions unique to each species. Therefore, sequencing hypervariable regions in or between rRNA genes is the basis of the phylogenetic analysis of bacteria and fungi. Conversely, this analysis can be used to identify bacteria or fungi in clinical specimens such as blood, fluid from sterile sites, and tissue, commonly referred to as broad-range PCR. The use of universal primers allows the untargeted testing of a vast range of bacteria and fungi. 

Broad-range PCR assays for blood are not readily available for clinical use but have been investigated in a few studies [16,17]. In a study by Flurin et al., the positivity of 16S rRNA gene PCR/sequencing for the whole-blood samples of patients with suspicion of IE was 65%, comparable to 71% positivity for mcfDNA sequencing [17]. Out of 28 positive blood culture cases, whole-blood 16S rRNA gene PCR/sequencing was positive for 17 (61%) [17]. As shown in this study, blood-based PCR assays can be falsely negative for a variety of reasons. Fida et al. hypothesized that the need for effective lysis of microbes, interference of human DNA, other inhibitory substances in blood, the short half-life of cell-free DNA, and the low concentration of microbes in blood could lead to the low sensitivity of blood-based broad-range PCR assays [18]. The detection of microbial contaminants and background noise in the DNA signals limit specificity [18].

The utilization of broad-range PCR assays for valve tissue can significantly reduce the number of microbiologically undefined cases in CNIE due to their higher sensitivity in antibiotic-exposed patients, detection of fastidious organisms, and higher specificity [19,20,21,22,23,24,25,26,27]. Due to these advantages, broad-range PCR assays for valve tissue have been widely employed in IE diagnosis over the past decade. Studies have reported a higher sensitivity of broad-range PCR assays for cardiac tissue (68% to 90%) compared with blood cultures and tissue cultures, and the difference in the positivity rate drastically increased in patients with prior antibiotic exposure [19,20,21,22,23,24,25,26,27]. The positivity rate of valve-tissue broad-range PCR assays further increased by 55% using next-generation sequencing compared with Sanger sequencing alone [19]. Multiple studies have reported the successful detection of fastidious organisms such as *Bartonella* spp., *Haemophilus parainfluenzae, Brucella* spp., *T. whipplei, C. burnetii,* and *Cutibacterium acnes* in broad-range PCR assays performed on cardiac tissue. Overall, studies have reported that 50% to 100% of CNIE cases had positive valve-tissue broad-range PCR results, demonstrating the value of performing broad-range PCR assays in addition to culture-based work-ups [19,20,21,22,23,24,25,26,27].

It is important to note that prolonged antibiotic exposure can decrease the yield not only of cultures but also of broad-range PCR testing. Halavaara et al. and Hong et al. showed that more than 2 weeks of preoperative antimicrobial therapy reduced the valve bacterial PCR positivity rate (53–67% PCR positivity for 2 weeks or more of preoperative antimicrobials versus 91–92% PCR positivity for less than 2 weeks of preoperative antimicrobials) [19,28].

Despite the considerable advantages of broad-range PCR testing, the assay is not readily available in all clinical microbiology laboratories. The laboratory should have the capacity to perform this sophisticated assay and computing power to run sequence analyses. In addition, the sequencing results should be interpreted by an expert microbiologist in the clinical context as differentiating between colonizing and pathogenic organisms might be challenging. Finally, broad-range PCR assays cannot provide phenotypic or genotypic information on the antimicrobial susceptibility of microorganisms.

## 5. Shotgun Metagenomic Sequencing

Shotgun metagenomic sequencing (sMGS) is an untargeted next-generation sequencing (NGS) approach performed by sequencing all fragmented DNA in a specimen, including microbial DNA. By far, it is the most unbiased method for microbe detection, and can theoretically detect any DNA-based microorganism, such as bacteria, fungi, DNA viruses, and parasites. 

sMGS using cardiac valve tissue is not available for routine clinical use. In studies, it has been shown to have superior sensitivity (86–100%) compared with blood or tissue cultures and was able to detect fastidious or unculturable microorganisms [29,30,31]. In contrast to one retrospective study that showed 100% sensitivity and specificity for tissue sMGS, a prospective study by Zeng et al. showed the specificity of tissue sMGS to be as low as 73% [29,31]. This was because only a small proportion (<1%) of the sequence reads were from non-human DNA, of which only some of the reads corresponded with potential pathogens [32]. sMGS using tissue is especially challenging to perform in individual clinical microbiology laboratories as there is more background human DNA than blood or other body fluid sources [32].

Plasma mcfDNA sequencing, also called a noninvasive “liquid biopsy”, is commercially available, with the Karius^®^ test (Redwood City, CA, USA) being one such assay. The assay can detect and report more than a thousand DNA pathogens and there is no need for invasive surgeries to obtain cardiac tissue. Previous studies showed that mcfDNA sequencing has high concordance with blood cultures and utility in CNIE, including in those exposed to antibiotics [33,34,35,36,37]. In addition, a Karius test reports the quantity of mcfDNA in molecules per microliter (MPM) and also reports important antimicrobial resistance genes in select pathogens. 

In the largest retrospective cohort (to date) of 141 patients in a tertiary hospital, mcfDNA sequencing identified the pathogen responsible for IE in 60.6% of definite or probable IE [38]. This was superior to routine work-up, including cultures, serologies, and targeted PCR assays, which had a positivity rate of 39.4% [38]. mcfDNA sequencing was the sole positive microbiologic test for 33.3% of IE cases, leading to an adjustment of antimicrobial therapy in 50% of those cases [38]. 

However, similar to tissue sMGS, common false positives or clinically insignificant detection remain important limitations, which makes any result subject to the interpretation of the clinician in distinguishing signal from noise [37,38,39]. Although false-negative cases may occur following prolonged antibiotic exposure (>7 days), mcfDNA can remain detectable significantly longer than conventional blood cultures (median 38.1 vs. 3.7 days), even with antibiotic therapy [33]. Notably, clinicians should be aware that important pathogens associated with IE may not be routinely detected by sMGS and mcfDNA sequencing [38]. For instance, *C. acnes* has been associated with prosthetic valve IE but has not been reported for conventional Karius testing. In addition, further prospective studies are needed to investigate how and when to utilize mcfDNA sequencing to maximize diagnostic and therapeutic benefits in a cost-effective manner.

## 6. Discussion

Molecular assays have become important diagnostic tools to solve microbiologically undefined IE cases. Table 1 summarizes and compares different molecular diagnostic techniques. In general, blood-based tests do not require invasive procedures or surgery to obtain cardiac tissue. However, blood-based tests have lower sensitivity than tissue-based tests. This is attributed to lower pathogen DNA concentrations in blood compared with tissue as well as a high quantity of PCR inhibitors present in blood. 

How could the utilization of different molecular assays be optimized for the diagnostic yield, adjustment of therapy, and favorable cost-benefit outcomes? The answer is unknown. For example, when there is no clear hypothesis for microbiology in the case of CNIE, whether the clinician should order a whole panel of pathogen-specific PCR assays, pursue metagenomic sequencing, or perform both is a difficult decision to make. Performing untargeted metagenomic testing early in the diagnostic work-up may lead to more cost-effective care by avoiding the need for multiple targeted tests and invasive procedures. In addition, due to the turnaround time of metagenomic testing, which is comparable to or even faster than a conventional work-up, a longer hospital stay or readmission due to delayed positive results could be prevented. 

The cost-effectiveness of shotgun metagenomic sequencing of blood has been investigated for other infectious syndromes such as invasive fungal infection (IFI) and fever of unknown origin (FUO) [37,43]. In a comprehensive cost-benefit analysis, MacIntyre et al. concluded that the introduction of the Karius test in the work-up of IFI was associated with a cost saving through its noninvasive approach [43]. In respect to FUO in immunocompromised patients, Ranganath et al. suggested potential cost benefits of the Karius test, especially in a subgroup of patients with an anticipated lowest diagnostic yield from conventional diagnostic tests [37]. A comprehensive cost-benefit analysis of the use of advanced molecular diagnostics in infective endocarditis is an important future direction.

**Figure 1:**
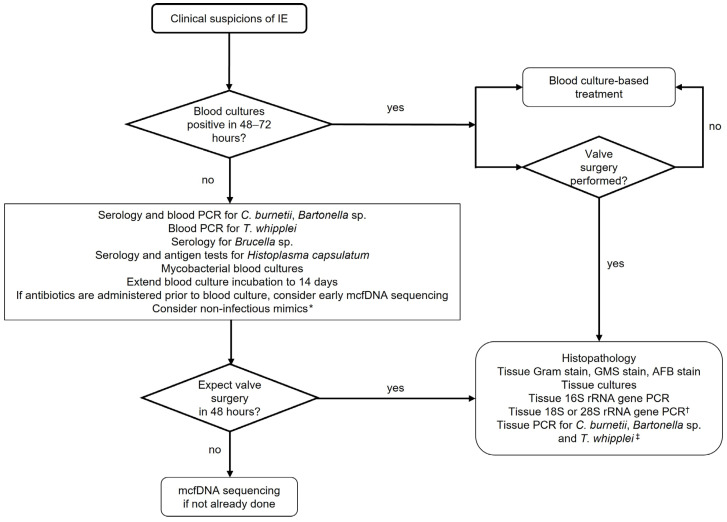
Suggested diagnostic approach for infective endocarditis. * Examples include nonbacterial thrombotic endocarditis, Libman–Sacks endocarditis, mechanical valve thrombosis, valve calcification, papillary fibroelastoma, and small vessel vasculitis. ^†^ If clinical suspicion of fungal endocarditis exists. ^‡^ For confirmatory purposes if blood-based pathogen-specific PCR or serology is positive or if clinical suspicion of a specific pathogen remains high. IE: infective endocarditis; PCR: polymerase chain reaction; *C. burnetii*: *Coxiella burnetii*; *T. whipplei*: *Tropheryma whipplei;* mcfDNA: microbial plasma cell-free DNA.

In Figure 1, we suggest a diagnostic approach for CNIE, considering diagnostic yield, testing availability, and the cost of each test. 

In a retrospective study at a large tertiary center, Kim et al. highlighted a subset of patients with “workup-negative IE” whose microbiological diagnosis could not be found, despite extensive culture-based, serological, and molecular studies. This shows the limitations of current IE diagnostics and the need for refined and innovative diagnostic technology.

For future directions, an ideal molecular test for IE would allow for noninvasive, blood-based, untargeted testing. This test would also be available for tissue-based testing. Ideally, both blood-based and tissue-based testing should have quick turnaround times and excellent sensitivity and specificity. Furthermore, quantitative measures of the pathogen and antimicrobial susceptibility results are desirable. 

## 7. Conclusions

A comprehensive diagnostic evaluation and a multimodal approach are crucial in diagnosing IE and guiding antimicrobial and surgical treatment. Molecular diagnostics could be a powerful tool adjunctive to cultures and histopathology to elucidate the microbiological etiology of IE, especially in culture-negative cases. However, there remains a group of “workup-negative IE” patients where exhaustive microbiological work-ups fail to unveil the pathogen, highlighting an area for future research and development. The utilization of various molecular diagnostics should be based on individual clinical scenarios as well as the test’s diagnostic strengths and weaknesses, availability, and cost. 

## Figures and Tables

**Table 1:** Comparison of molecular diagnostic assays for the diagnosis of infective endocarditis.

Assay	Specimen	Examples	Available for Clinical Use	Advantages	Limitations	Sensitivity and Specificity	References
Pathogen-specific PCR	Blood; tissue	*C. burnetii* PCR, *Bartonella* PCR, and *T. whipplei* PCR	Yes	- Less expensive - High sensitivity in tissue	- Low sensitivity in blood/serum	Sn: 33–69% (blood); 25–72% (tissue) Sp: ~100%	[4,5,6,7,8,9,10]
Multiplex RT-PCR	Tissue	BIOFIRE^®^ Blood Culture Identification Panel ^a^; Unyvero Implant and Tissue Infection ^b^	No	- Fast time to result - Maintains sensitivity in prior antibiotic use - Can detect select resistance gene markers	- Only detects limited number (25–30) of pathogens	Sn: 60–100% Sp: ~100%	[11,12,13,40,41]
Broad-range PCR	Blood	16S rRNA gene PCR/sequencing; 18S or 28S rRNA gene PCR/sequencing	No	- Untargeted detection - Maintains sensitivity in prior antibiotic use	- No information for AST	Sn: ~60% Sp: not reported in the literature	[16,17]
	Tissue		Yes	- High sensitivity - Untargeted detection - Maintains sensitivity in prior antibiotic use	- No information for AST - High cost	Sn: 70–90% Sp: >90%	[19,20,21,22,23,24,25,26,27,28,42]
Shotgun metagenomic sequencing (sMGS)	Blood	Plasma mcfDNA sequencing (Karius^®^ test)	Yes	- Untargeted detection - Maintains sensitivity in prior antibiotic use (overall sensitivity 60–70%) - Can detect select resistance gene markers	- Detection of clinically insignificant organisms - Subjectivity in clinical interpretation - Commercial tests do not report certain organisms, including common skin bacteria - High cost	Sn: 60–70% Sp: ~70%	[33,34,35,36,38]
	Tissue	Tissue sMGS	No	- Untargeted detection - Maintains sensitivity in prior antibiotic use - Can detect select resistance gene markers	- Detection of clinically insignificant organisms - Subjectivity in interpretation - High cost	Sn: 86–100% Sp: 73–100%	[29,30,31,32]

^a^ bioMérieux, Inc. Marcy-I’Étoile, France. ^b^ Curetis GmbH, Holzgerlingen, Germany. PCR: polymerase chain reaction; Sn: sensitivity; Sp: specificity; RT-PCR: real-time PCR; *C. burnetii*: *Coxiella burnetii*; *T. whipplei*: *Tropheryma whipplei;* AST: antimicrobial susceptibility; mcfDNA: microbial plasma cell-free DNA.

## Data Availability

No new data were created or analyzed in this study. Data sharing is not applicable to this article.
